# OM2, a Novel Oligomannuronate-Chromium(III) Complex, Promotes Mitochondrial Biogenesis and Lipid Metabolism in 3T3-L1 Adipocytes via the AMPK-PGC1α Pathway

**DOI:** 10.1371/journal.pone.0131930

**Published:** 2015-07-15

**Authors:** Jiejie Hao, Cui Hao, Lijuan Zhang, Xin Liu, Xiaolin Zhou, Yunlou Dun, Haihua Li, Guangsheng Li, Xiaoliang Zhao, Yuanyuan An, Jiankang Liu, Guangli Yu

**Affiliations:** 1 Key Laboratory of Marine Drugs, Ministry of Education, Ocean University of China, 5 Yushan Road, Qingdao, 266003, China; 2 Shandong Provincial Key Laboratory of Glycoscience and Glycotechnology, Ocean University of China, Qingdao, 266003, China; 3 Center for Mitochondrial Biology and Medicine, The Key Laboratory of Biomedical Information Engineering of Ministry of Education, School of Life Science and Technology and Frontier Institute of Science and Technology, Xi'an Jiaotong University, Xi'an, 710049, China; Northeast Ohio Medical University, UNITED STATES

## Abstract

**Background:**

In our previous studies, we prepared novel oligomannuronate-chromium(III) complexes (OM2, OM4) from marine alginate, and found that these compounds sensitize insulin action better than oligomannuronate(OM), chromium, and metformin in C2C12 skeletal muscle cells. In the present study, we studied their effects on mitochondrial biogenesis, lipid metabolism, and the underlying molecular mechanisms in differentiated 3T3-L1 adipocytes.

**Methodology/Principal Findings:**

We firstly used the pGL3-PGC1α and pGL3-ATGL promoter plasmids to compare their effects on PGC1α and ATGL transcription activities. Then mitochondrial biogenesis was quantified by transmission electron microscopy and MitoTracker staining. Mitochondrial oxygen consumption and fatty acid oxidation were measured by an oxygen biosensor system and ³H-labelled water scintillation. The mitochondrial DNA and mRNA involved in mitochondrial biogenesis and lipid oxidation were evaluated by real-time PCR. AMPK together with other protein expression levels were measured by western blotting. The inhibitor compound C and siRNA of PGC1α were used to inhibit the OM2-induced AMPK-PGC1α signaling pathway. And we found that OM2 stimulated AMPK-PGC1α pathway in the 3T3-L1 adipocytes, which were correlated with induced mitochondrial biogenesis, improved mitochondrial function, and reduced lipid accumulation by enhanced fatty acid β-oxidation and augmented ATGL protein expression.

**Conclusions/Significance:**

Our data indicated that the marine oligosaccharide-derived OM2 might represent a novel class of molecules that could be useful for type 2 diabetes prevention and treatment by up-regulating AMPK-PGC1α signaling pathway.

## Introduction

The World Health Organization estimates that 180 million people have been afflicted and that the number will double by 2030. The medications used based on current scientific knowledge are insufficient to prevent/cure type 2 diabetes. New anti-diabetic agents that prevent and reduce insulin resistance, hyperglydemia, and hyperlipidemia are needed to combat this disease.

Mitochondria play central roles in energy homeostasis, metabolism, signaling, and apoptosis [1]. Clinical studies of obesity patients with insulin-resistant type 2 diabetes show that mitochondrial functions are declined, which are associated with a reduction of both mitochondrial DNA (mtDNA) copy numbers and key factors regulating mitochondrial biogenesis [2]. Impaired mitochondrial biogenesis and functions in adipose tissue are also observed in animal models of type 2 diabetes [3–5]. Either life style interventions (i.e. exercise and calorie restriction) or pharmacological treatments (i.e. thiazolidinediones or metformin) increase oxidative metabolism in mitochondria and enhance whole body insulin sensitivity. The enhanced insulin sensitivities are correlated with mitochondrial biogenesis and enhanced mitochondrial functions in cultured adipocytes, skeletal muscles, and diabetic volunteers [6–9]. However, it is unknown if the enhanced insulin sensitivities lead to enhanced mitochondrial functions and biogenesis or visa versa, but enhancing insulin sensitivities and mitochondria functions plus promoting mitochondrial biogenesis are common goals for prevention and treatment of both type 2 diabetes and obesity [10].

Central to mitochondrial biogenesis and enhanced mitochondrial function is the activation of peroxisome proliferator-activated receptor gamma coactivator-1 alpha (PGC1α). PGC1α targets multiple specific transcription factors, leading to replication of mtDNA and expression of mitochondrial proteins to stimulate mitochondrial metabolic capacity and function [11]. One major regulator upstream of PGC1α is AMP-activated protein kinase (AMPK), which serves as a “fuel gauge” in cells and plays an important role in metabolic function. AMPK acts in concert with the PGC-1α to regulate energy homeostasis in response to environmental and nutritional stimuli, representing the most important signaling pathway in mitochondrial biogenesis [12, 13].

In our previous studies, we prepared novel oligomannuronate-chromium(III) complexes (OM2, OM4) from marine alginate, and found that these compounds sensitize insulin action better than oligomannuronate, chromium, and metformin in C2C12 skeletal muscle cells. These compounds also have lower toxicity profile than that of metformin [14].

Compared with skeletal muscle, adipose tissue plays an equivalent or more important role in the progress of obesity and diabetes for its direct involvement in metabolic and endocrinal regulations [15]. Excessive fat accumulation in the white adipose tissue causes obesity and results in an increased risk for many serious diseases, including type 2 diabetes, hypertension, and heart diseases [16]. In addition, lipolysis plays a pivotal role in controlling the quantity of triglycerides stored in fat tissue and free fatty acid levels in plasma. Recent data from different laboratories clearly demonstrate that adipose triacylglycerol lipase (ATGL), a newly discovered lipase, which catalyses the hydrolysis of the first ester bond of stored triacylglycerol, is an important rate-limiting factor in triacylglycerol hydrolysis [17, 18]. Therefore activators of lipolysis through enhanced ATGL function also attract great pharmacological interest [19].

In the present study, We demonstrated that OM2 stimulated AMPK-PGC1α pathway in the 3T3-L1 adipocytes, which were correlated with induced mitochondrial biogenesis, enhanced mitochondrial function, and reduced lipid accumulation by enhanced fatty acid β-oxidation and augmented ATGL protein expression. Our data indicate that OM2 might exert beneficial effects for type 2 diabetes patients associated with dyslipidemia and obesity.

## Materials and Methods

### Materials

The marine-alginate derived oligomannuronate and its chromium (III) complexes named OM, OM2 (with 2% chromium), OM4 (with 4% chromium), were prepared as previously described [14]. The weight-averaged molecular masses of OM, OM2 and OM4 were 2.8, 3.0 and 3.2 kDa, respectively, measured by high performance gel permeation chromatography. The chromium (III) chloride was from Sinopharm ChemicalInc. Anti-tubulin was from Sigma (St. Louis, MO, USA); anti-PGC1α was from Santa Cruz; anti-AMPK (Thr172), anti-ACC (ser 72) and anti-ATGL were from Cell Signaling Technology; anti-OxPhos Complex I (NADH ubiquinol oxidoreductase 39 kD subunit), and anti-OxPhos Complex II (succinate-ubiquinone oxidoreductase 70kD subunit) were from Invitrogen; Reverse Transcription System kit was from Promega; HotStarTaq was from Takara; Metformin was a gift from Taian Pharmaceutical Co., Ltd (Shandong, China). TRIzol and other reagents were from Invitrogen (Carlsbad, USA).

### Cell culture and reporter assays

Murine 3T3-L1 pre-adipocytes (American Type Culture Collection) were cultured and differentiated into fully mature adipocytes as previously described [20]. A ∼2kb PGC1α promoter in pGL3-basic luciferase reporter construct and a ∼3kb ATGL promoter in pGL2-basic luciferase reporter construct were gifts from Dr. X. Ge (Shanghai Institutes of Biological Sciences, Shanghai, China). Fully differentiated cells were transfected with pGL3-PGC1α or pGL3-basic plasmid (pGL2-ATGL or pGL2-basic plasmid) using the Cell Line Nucleofector Kit from Amaxa (Gaithersburg, MD) following the manufacturer's instructions. The Rellina vector was used to monitor the transfection efficiency. The transfected cells were cultured for 18 hours and then incubated with indicated concentrations of OM and its complexes or chromium III for 24 hours. The reporter activity was measured by a luciferase assay kit (KenReal, Shanghai, China) with a luminometer (Berthold Technologies, BadWildbad, Germany). The relative Luc activity was calculated as the ratio of firefly Luc activity to Rellina luc activity. Transfections were performed in duplicate and repeated four times.

### Western blot analysis

Cell lysates were subjected to electrophoresis through 10% SDS-PAGE and blotted with antibodies for PGC1α, ATGL, AMPK, phosphor-AMPK, acetyl-CoA (ACC), phosphor-ACC, complex I and complex II. The β-actin or α-tubulin was used as control. The immunoblots were visualized by chemiluminescence.

### Mitochondria isolation, RNA isolation and real-time PCR

The mitochondria were isolated by differential centrifugation of the cell homogenates as previously described [20]. Gene expression was analyzed by real-time PCR (RT-PCR). Total RNA prepared from the 3T3-L1 adipocytes was extracted with Trizol (Ivitrogen). Complementary DNA generated by M-MLV Reverse transcriptase (Promega) was analyzed by quantitative PCR using an SYBR *Premix Ex Taq* (TaKaRa). mRNA expression of target gene was normalized to the internal control 18S rRNA and quantified.

### Cell respiration test and Nucleotide study

Oxygen consumption by intact cells was measured as an indication of mitochondrial respiration activity. The BD Oxygen Biosensor System utilizes an oxygen-sensitive fluorescent compound (Tris 1,7-diphenyl-1,10 phenanthroline ruthenium [II] chloride) embedded in a multi-well plate. The fluorescence correlates directly to oxygen consumption. After treated with different concentrations of testing compounds, the adipocytes were washed and measured as described [20]. Results are expressed as the slope of time-varying fluorescence intensity. V_max_ is the maximum consumption rate. The cellular levels of ATP, ADP and AMP were measured in protein-free extracts prepared as follows. 0.6mol·L ^-1^ perchloric acid (ice-cold) was used to lyse cells and precipitate proteins. After centrifugation, the supernatant was neutralized with KOH (2mol·L ^-1^) and MOPS (0.3mol·L ^-1^). Then the supernatants were stored at -80°C and used for HPLC-based nucleotide quantification as described [21].

### Mitochondrial mass assay

The fluorescent probe MitoTracker Green FM (Molecular Probes, Eugene, OR) was used to determine the mitochondrial mass of adipocytes. In brief, the treated adipocytes were incubated with 0.1 μmol/l MitoTracker Green FM in KRH buffer for 30 min at 37°C, then were centrifuged, resuspended and fluorescence was analyzed by flow cytometry (FACS Calibur, Becton Dickinson, Mountain View, CA).

### Electron microscopy

The electron microscopic photographs were taken as described [20], and analyzed blindly. For each individual adipocyte profile in the area, the numbers of mitochondria were counted, and the area of total mitochondrial sections were analyzed. Measurements were made on 10 individual adipocytes for each type of treatment.

### Palmitate β-oxidation and triglyceride measurement

The 3T3-L1 cells in 6-well culture plates were resuspended in medium containing 2 μCi [9,10-^3^H]palmitic acid, After 24 h, the supernatant was applied to an ion-exchange column (Dowex 1×8–200; Sigma, St Louis, MO, USA), and ^3^H-labelled water was recovered by elution with 2.5 ml water. A 0.6 ml aliquot was then used for scintillation counting, and the values were expressed in the absolute units of the assays (nmol· h^-1^·mg protein ^-1^). The cellular triglyceride measurement were performed according to the method of Shimabukuro et al. [22], and results were expressed as microgram triglyceride per milligram of cellular protein.

### Transfection of siRNA

3T3-L1 cells were fully differentiated as described [20]. The differentiated adpocytes in six-well plates were transfected with siRNAs for PGC1α and control, respectively, using siRNA Transfection Reagent according to the manufacturer’s instructions (Santa Cruz Biotechnology, CA, USA). After media change, the transfected cells were then treated with or without OM and OM2 for 24 h.

### Statistical analysis

All data are expressed as means ± SEM. Differences were analyzed by one-way ANOVA. A value of p<0.05 was accepted as statistically significant.

## Results

### Stimulated gene transcription and protein expression of PGC1a- and ATGL—in the adipocyte

**Fig 1:**
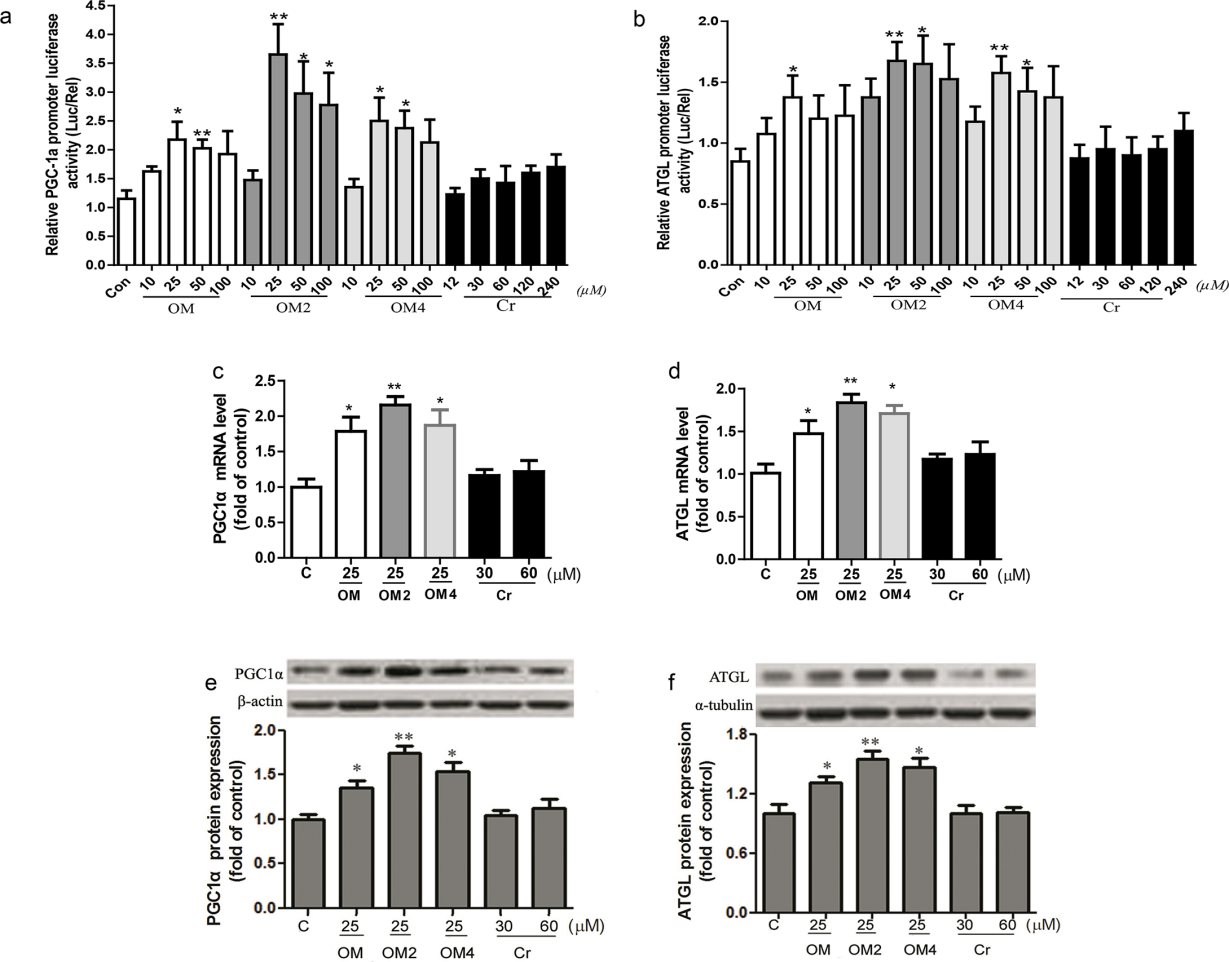
OM2 Stimulated gene transcription and protein expression of PGC1a and ATGL. Fully differentiated cells were transfected as described above and incubated with different concentrations of OM and its complexes or chromium III for 24 hours. The reporter activity of PGC1a (a) and ATGL (b) were measured by a luciferase assay kit with a luminometer; total RNA of the adipocytes were isolated and analyzed by quantitative RT-PCR with gene-specific oligonucleotide probes for mRNA of PGC1α (c) and ATGL (d). The cycle number at which the various transcripts were detectable was compared with that of 18s rRNA as an internal control; as for protein expression detection, the adipocytes in 6-well plates were treated and subsequently solubilized into SDS sample buffer and analyzed by western blotting with antibodies against β-actin or ɑ-tubulin and objective antibodies. (e) representative of PGC1α protein immunoblots *(upper)* and quantitative analyzes *(lower)*; (f) representative of ATGL protein immunoblots *(upper)* and quantitative analyses by densitometry *(lower)*. Results are presented as percentage of untreated control cells. Values are mean ± SEM (n = 4). *p<0.05, **p<0.01 vs control.

PGC1α is a key factor that drives mitochondrial biogenesis and stimulates fatty acid oxidation in muscle and adipose tissues. So in the present study, we firstly used the pGL3-PGC1α promoter plasmid to compare the effects of OM and its complexes or chromium III on PGC1α transcription activity. As shown in Fig 1A, treatment of the adipocyte with the marine acidic oligosaccharides OM, OM2 and OM4 at 10–100μmol·L ^-1^ resulted in a dose-dependent stimulation of PGC1α promoter transcription by luciferase assay. OM2 significantly elevated PGC1α promoter transcription activity at 25–100 μmol·L ^-1^, while the significant stimulation effect of OM4 on the transcription activity occurred at 25–50μmol·L ^-1^. The marine acidic oligosaccharides OM increased ∼1.8 fold of PGC1α transcription activity, whereas OM2 and OM4 increased its activity by ∼3.1 fold and ∼2.2 fold at 25μmol·L ^-1^, respectively as compared with the control. Meanwhile, the corresponding equivalent concentrations of chromium III from 12 to 240μmol·L ^-1^ did not significantly affect the activity, only tended to increase at concentrations above 120μmol·L ^-1^. Moreover, Fig 1A showed that the effective concentration of oligomannuronate and its chromium III complexes to stimulate PGC1α transcription activity was optimized at 25–50μmol·L ^-1^, and OM2 was the most effective compound among the oligosaccharides.

Then we investigated whether they could regulate the ATGL transcriptional level or not by luciferase assay which representative of initial lipolysis. The data in Fig 1B showed that treating the adipocyte with either OM and its chromium III complexes resulted in bell-shaped response curves of ATGL promoter transcription stimulation, which was similar to that of PGC1α promoter transcription induction. OM induced the transcription activity significantly at 25 μmol·L ^-1^, while the increases of OM2 and OM4 were most significant at 25–50 μmol·L ^-1^. The activity was increased ∼0.6 fold by OM at optimized concentration of 25μmol·L ^-1^ as compared with control, and was increased ∼2.0 and ∼1.8 fold by OM2 or OM4 at optimized 25μmol·L ^-1^, respectively. Whereas the activity did not change significantly by equivalent concentrations of chromium III treatment (Fig 1B), and obviously OM2 was also the most effective compound among the oligosaccharides, which was consist to the PGC1α transcription activity assay.

PGC1α and ATGL mRNA and protein expression levels were also significantly increased by OM, OM2 and OM4 at 25μmol·L ^-1^ but not by chromium (III) at 30μmol·L ^-1^ to 60μmol·L ^-1^ concentration range (Fig 1C–1F). All the results shown in Fig 1 suggested that OM and its chromium complexes but not chromium (III) played a major role in the up-regulated expression of PGC1α and ATGL proteins.

### Increased mitochondrial biogenesis and functions in relation to critical mitochondrial proteins, mRNA, and mtDNA levels in the adipocytes

Based on the data shown in Fig 1A and 1D, the optimal effect of stimulation was found to be at 25–50μmol·L ^-1^. Thus, the adipocyte was treated with OM and OM2 at concentrations of 25μmol·L ^-1^ or 25–50μmol·L ^-1^.

**Fig 2:**
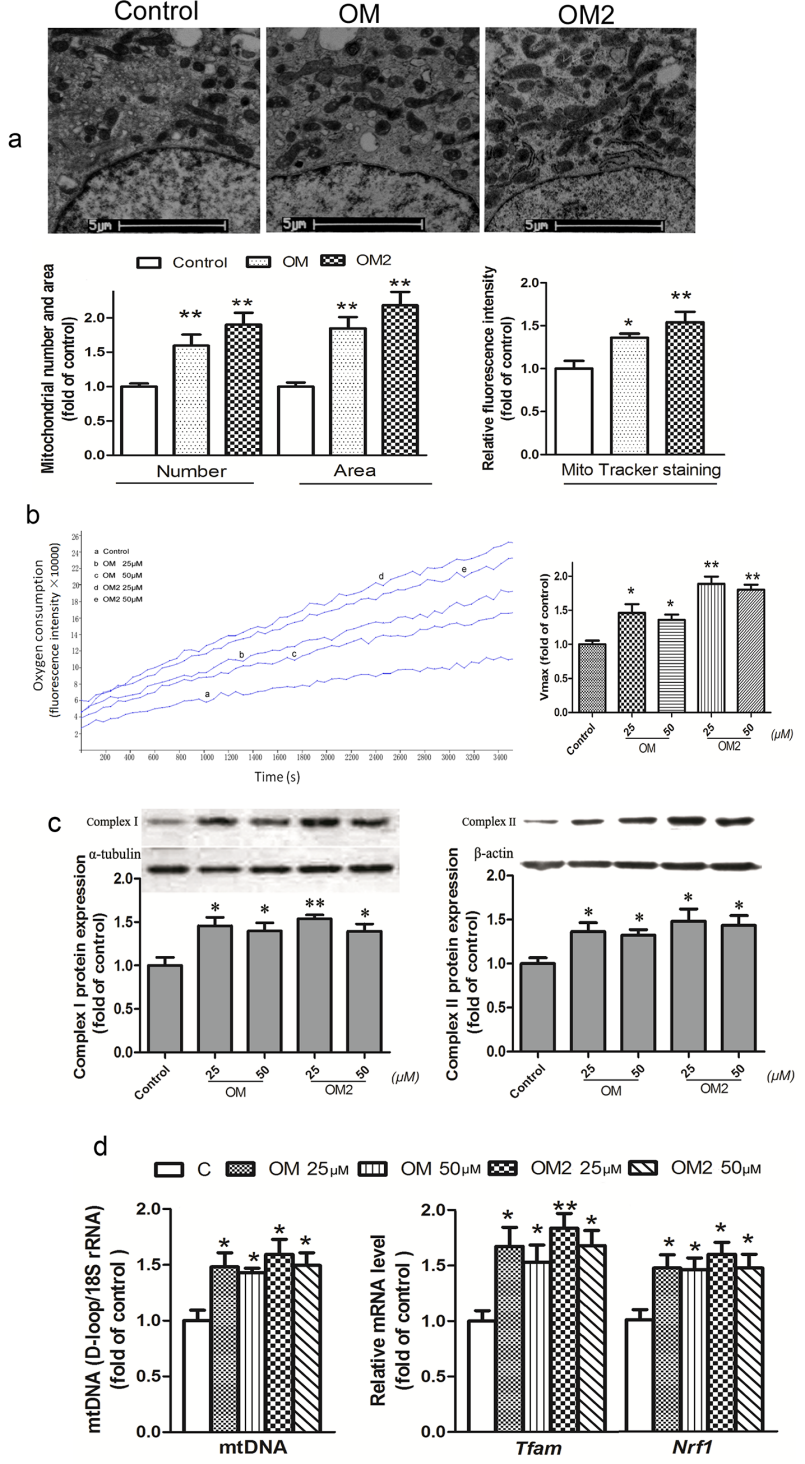
OM2 promoted mitochondrial biogenesis and function. The fully differentiated adipocytes were treated or untreated for 24 h before analysis; (a) *upper*, the adipocyte mitochondrial morphometry illustrated by transmission electron microscopy; *lower*, quantitative analysis of mitochondrial density and surface area by transmission electron microscopy and MitoTracker Green FM staining (100 nmol/l); (b) oxygen consumption in that equal volumes of cells were separated into aliquots in wells of a 96-well BD Oxygen Biosensor plate. Fluorescence in each well was recorded over time. *Left*, representative oxygen consumption curves; *Right*, quantitative changes in the respiratory rate of the adipocytes were calculated by determining the kinetic measurements; (c) *upper*, a representative immunoblot of complex I and II; *lower*, quantitative analysis by densitometry; (d) mtDNA levels and mRNA levels of *Nrf1* and *Tfam*, total RNA or DNA of the adipocytes were isolated and analyzed by quantitative RT-PCR with gene-specific oligonucleotide probes. The cycle number at which the various transcripts were detectable was compared with that of 18s rRNA as an internal control. All values are mean±SEM of four independent experiments. *p<0.05, *p<0.01 vs control.

Quantitative statistical data of mitochondrial number and size in individual adipocytes obtained by transmission electron microscopy (Ten cells were analyzed for each type of treatment) were given in Fig 2A after 48 h treatment. The data indicated that OM2 and OM at 25μmol·L ^-1^ caused significant mitochondrial biogenesis compared to that of the control cells. We also used MitoTracker Green FM to label and quantify mitochondria in the adipocytes (Fig 2A). The two independent measurements showed that OM2 and OM at 25μmol·L ^-1^ increased mitochondrial number, area, and MitoTracher stain by ∼1.4 to ∼2.1 fold compared to that of the control.

We next measured if the OM2 induced mitochondrial biogenesis correlated with enhanced mitochondrial functions. Fig 2B indicated that OM and OM2 treatment at 25 and 50μmol·L ^-1^ elevated oxygen consumption by ∼1.3 to ∼1.9 fold compared with that of the control. As shown in Fig 2C, treatment of OM and OM2 for 48 h at 25∼50μmol·L ^-1^ also significantly increased the expression of mitochondrial respiratory Complex I and Complex II by ∼1.3 to ∼1.6 fold compared to that of the control, which associated with an elevation in mtDNA copy number and mitochondrial transcriptional nuclear factors *Nrf1* and *Tfam* mRNA expression (Fig 2D). All these results presented in Fig 2 showed that the OM2 induced mitochondrial biogenesis and enhanced mitochondrial functions, which were associated not only with increased mtDNA copy numbers but also with critical transcription factor expression levels.

### Increased fatty acid β-oxidation in the adipocytes

**Fig 3:**
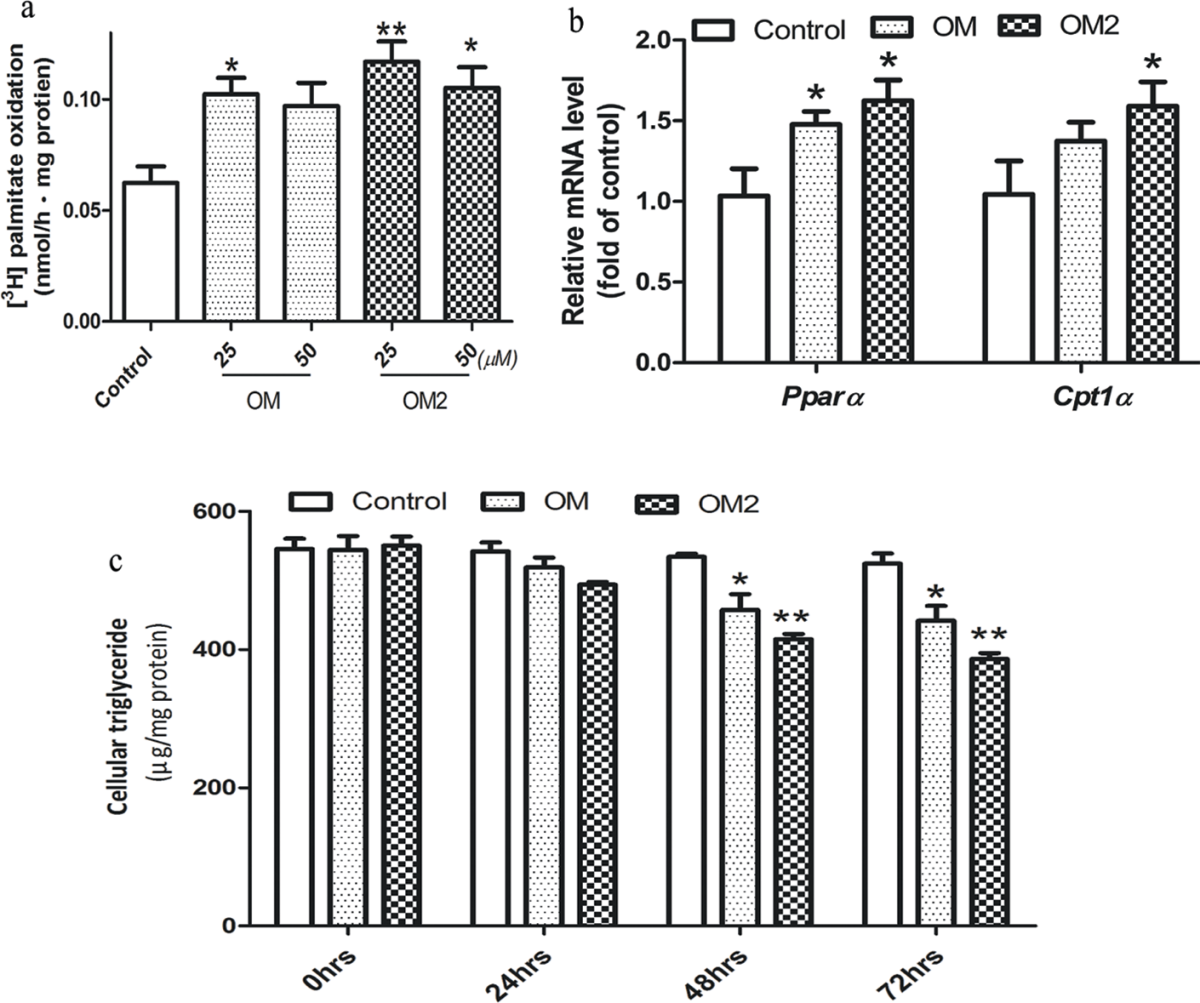
OM2 improved lipid metabolism. (a) The adipocytes treated or untreated for 24 h were then measured for [^3^H] palmitate oxidation as described in the method section; (b) mRNAs of lipid metabolism genes *Pparα* and *Cpt1α* were analyzed by quantitative RT-PCR with gene-specific oligonucleotide probes. The cycle number at which the various transcripts were detectable was compared with that of 18s rRNA as an internal control; (c) the intracellular triglycerides were measured and expressed as μg of lipid per mg of protein. Results were expressed as percentage of untreated control cells. Values are mean±SEM of the results from three independent experiments. *p<0.05, **p<0.01 vs control.

It was reported that chemicals stimulated mitochondrial biogenesis is associated with increased fatty acid β-oxidation in the 3T3-L1 adipocytes [23], we performed the ^3^H-palmitate β-oxidation experiment. The adipocyte was treated with OM and OM2 at the optimal concentrations of 25 and 50μmol·L^-1^. We found that OM and OM2 treatment significantly increased the rate of β-oxidation at both 25 and 50μmol·L ^-1^ (Fig 3A). And the mRNA levels of fatty acid oxidation-related genes *Pparα* and *Cpt1α* were increased accordingly (Fig 3B). To investigate whether the elevated fatty acid β-oxidation would change the lipid accumulation, we tested the content of triglyceride in 3T3-L1 adipocytes after treating the cells with OM or OM2 for 0–72 h. As shown in Fig 2C, intracellular triglyceride accumulation was reduced by both OM and OM2 treatment in a time-dependent manner in that OM2 lowered triglyceride content by 30% and OM by 18% compared to that of control at 72 h (Fig 3C).

### AMPK-PGC1α pathway was required for OM2-mediated effect

**Fig 4:**
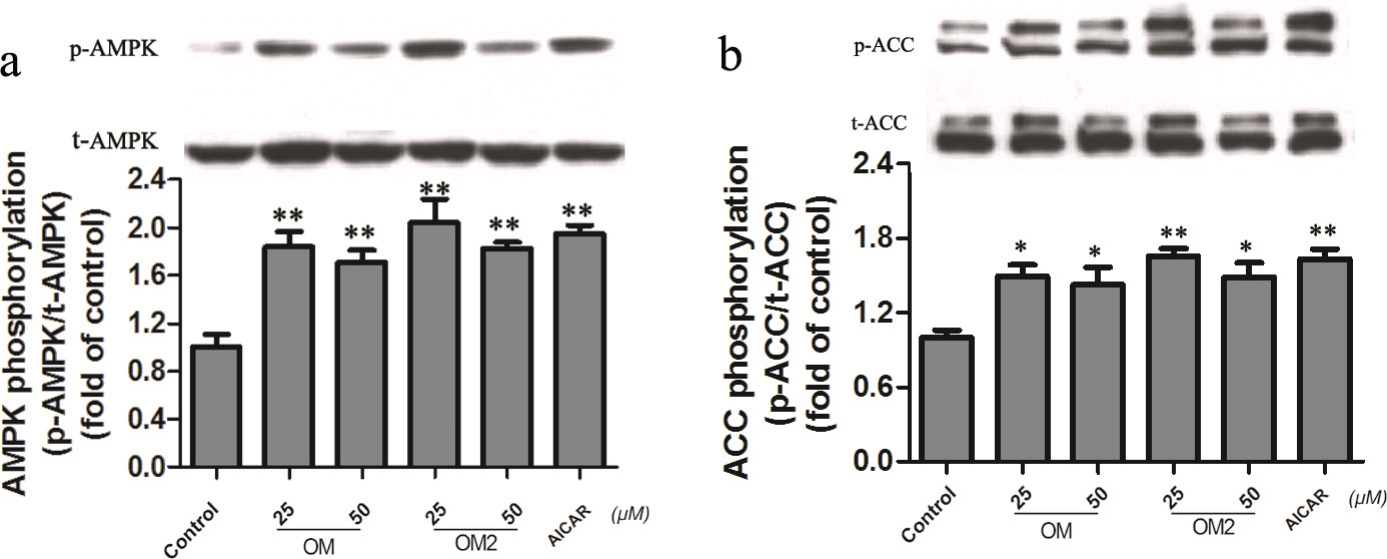
Effect of OM2 and OM on AMPK activation. The adipocytes were treated or untreated for 24 h, the cells were subsequently analyzed by western blotting for AMPK and ACC. a: *Upper*, a representative immunoblot of p-AMPK and t-AMPK; *Lower*, quantitative analysis of the ratio of p-AMPK/ t-AMPK; b: *Upper*, a representative immunoblot of p-ACC and t-ACC; *Lower*, quantitative analysis of the ratio of p-ACC / t-ACC. Values are mean±SEM of the results from three independent experiments. *p<0.05, **p<0.01 vs control.

AMPK acts as an energy sensor to regulate mitochondrial biogenesis, glucose and lipid metabolism [13, 24], therefore we tested if the OM2 treatment of 3T3-L1 adipocytes would activate the AMPK signaling pathway, which was done by measuring the level of phosphorylation of AMPK and ACC, a downstream target of AMPK. To be consistent, the 3T3-L1 adipocyte was also stimulated with OM and OM2 at the optimized concentration of 25∼50μmol·L ^-1^. As shown in Fig 4, significant activation of AMPK by OM and OM2 at 25∼50μmol·L ^-1^ was accompanied by increased phosphorylation of ACC. Moreover, OM2 at 25μmol·L ^-1^ induced a similar level AMPK and ACC phosphorylation as positive control AICAR at 1mmol·L ^-1^, indicating that OM2 was a potent AMPK activator.

**Fig 5:**
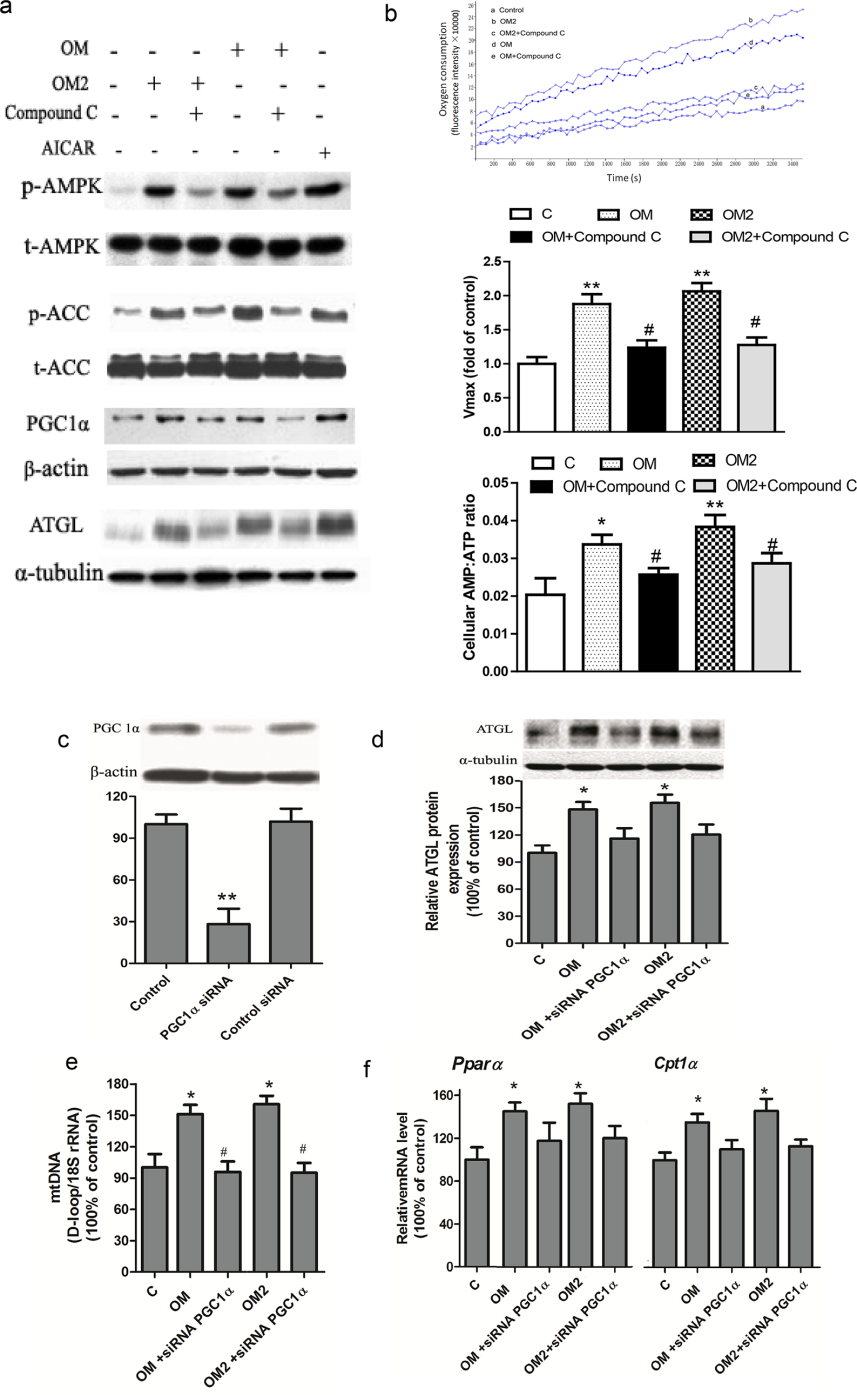
OM2 activated AMPK-PGC1a signaling pathway. (a-b) the adipocytes were pre-incubated with compound C (20 μmol/l) for 1 h, then were treated or untreated for 24 h; (a) a representative western blotting result of the indicated proteins of p-AMPK and p-ACC, PGC1α and ATGL. AICAR (1 mmol/l) was used as a positive control; (b) Oxygen consumption and AMP: ATP ratio of the adipocytes with indicated treatmen. *Upper* is representative oxygen consumption curves; *Middle* is quantitative changes in the respiratory rate; *Lower* is ratio changes in AMP/ATP; (c-f) the adipocytes were transfected with PGC1α siRNA for 48 h, then were treated or untreated for 24 h; (c) a representative immunoblot of PGC1ɑ *(upper)* and quantitative analysis *(lower)*; (d) a representative immunoblot of ATGL *(upper)* and quantitative analysis *(lower)*; (e) mtDNA content; (f) mRNA levels of *Pparα* and *Cpt1α*. Values are mean ± SEM from three independent experiments. *P < 0.05, **P < 0.01 versus control; #*P* < 0.05, ##*P* < 0.01 versus OM2 or OM treated group.

Thus far, we observed that OM2-induced mitochondrial biogenesis and lipid metabolism through transcriptional activation of PGC1α and activation of AMPK pathway. To test if the transcriptional activation of PGC1α was the results of the activated AMPK pathway by the OM2 treatment, we decided to use the compound C to inhibit AMPK activation and use siRNA of PGC1α to down-regulate PGC1α to understand the relationship between AMPK and PGC1α. As shown in Fig 5A, OM or OM2 dramatically increased the AMPK and ACC phosphorylation, which were accompanied by increased in PGC1α and ATGL protein expression. However, these effects were significantly blocked by compound C, suggesting that AMPK acted upstream of PGC1α. And it was well reported that compound C could block the effect of ALCAR on AMPK activation and the down-stream signal transduction, which was partly similar to the action mode of OM or OM2 observed in our study. Indeed, the enhanced oxygen consumption and AMP: ATP ratios induced by OM or OM2 were also abolished by pretreatment with the AMPK inhibitor compound C (Fig 5B). It has been known that compound c could not affect the ratio of AMP/ATP triggered by mitochondria function modulation [25, 26]. The blocking effect of compound C on AMP: ATP ratios observed in our study might be resulted from the feedback of the mitochondria during the chronical treatment of the adipocytes with OM or OM2. The compound C was reported to inhibit many kinases in addition to AMPK despite its common use. Therefore, specific AMPK inhibition by using AMPK siRNA or shRNA model would be ideal to confirm the observation in near future.

We then performed PGC1α siRNA-knockdown studies in the adipocytes. As shown in Fig 5C, introducing siRNA-PGC1α to the cells dramatically decreased PGC1α protein levels. Next, we found the OM or OM2 up-regulated ATGL protein expression was significantly abolished by pretreatment of the adipocytes with PGC1α siRNA (Fig 5D), Meanwhile, PGC1α siRNA treatment also blocked the elevated *Pparα* and *Cpt1α* mRNAs and mtDNA copy number induced by OM or OM2 (Fig 5E and 5F), indicating that PGC-1α played an importance role in fatty acid oxidation and mitochondrial biogenesis. All of these observations demonstrated that OM or OM2 affected in an AMPK-PGC1α pathway-dependent manner.

## Discussion

It has been reported that different poly- or oligosaccharides exert anti-diabetic activities, such as chitin-based oligosaccharides and its derivatives [27, 28], fructo-oligosaccharides [29], and others [30]. However, the molecular mechanisms remain unknown. In the present study, we demonstrated that OM, OM2, and OM4 stimulated AMPK- PGC1α pathway in the 3T3-L1 adipocytes, which were correlated with induced mitochondrial biogenesis, enhanced mitochondrial function, and reduced lipid accumulation by enhanced fatty acid β-oxidation. Our data suggested that promoting mitochondrial biogenesis and lipid metabolism via the AMPK-PGC1α pathway might underlie the reported anti-diabetic activities of poly- and oligosaccharides.

Chromium (III) is known for its beneficial role in carbohydrate and lipid metabolism [31]. Several oligosaccharides-chromium (III) complexes have been studied for their role in attenuating metabolic disturbances in diabetes [31–33]. Alginate oligosaccharide is regarded as a non-toxic, non-immunogenic polymer, making it an attractive candidate for treating inflammation [34], cancer [35], neurodegenerative disease [36], and other disaeses [37, 38]. Furthermore, chemically sulfated alginate-oligosaccharide showed better anti-diabetic activities [39, 40]. In our present studies, we showed for the first time that the novel alginate-derived oligomannuronate-chromium(III) complex, OM2, induced mitochondrial biogenesis and enhanced lipid metabolism, better than that of OM, and OM had better effect than that of chromium(III). These studies paved the way for potential applications of OM or its chromium complex, OM2, in type 2 diabetes prevention and treatment.

We previously reported that OM2 has better effect than the original oligosaccharide OM on insulin sensitivity in C2C12 cells [14]. Consistently, OM2 induced higher levels of PGC1α and ATGL protein expressions than the original oligosaccharide OM in the adipocytes (Fig 3). These results indicated that the introduction of chromium (III) to the oligosaccharides OM elevated the activity towards the PGC1α and ATGL activation. However, the activity of OM4 was lower than that of OM2 although it has 4% (w/w) chromium (III). These observations indicated that higher level of chromium (III) in the oligosaccharide complex was not correlated with higher activities. Moreover, chromium alone did not show much activity, which suggests the oligosaccharide and its chromium (III) complex, but not chromium (III), were the major contributors to the observed activities.

Mitochondrial biogenesis could, in part, underlie the central role of adipose tissue in the control of whole-body metabolism and that the loss of mitochondria in adipose tissue is correlated with the development of type 2 diabtetes. Herein, our results indicated that the anti-diabetic effect of OM2 was closely associated with or largely depended on stimulation of PGC1α-mediated mitochondrial biogenesis in the adipocytes. The activation of PGC1α is associated with a number of signaling pathways involving the activation of AMPK, intracellular calcium, the subsequent activations of calcium-sensitive calcium/calmodulin-dependent protein kinase, nitric oxide, and cAMP-responsive element binding protein signaling pathways [13, 41, 42]. OM2 stimulated the AMPK signaling pathway was evidenced by increased phosphorylation of AMPK and ACC (Fig 4), however, further studies are required to find out if OM2 also affects other signaling pathways related to AMPK-PGC1α activation.

Mitochondrial fatty acid β-oxidation is one of the key processes in energy production. The potent effects of OM2 on phosphorylation of AMPK and ACC and subsequent mitochondrial biogenesis and remodeling, were in accordance with the increases in mRNA of *Cpt1α* and *Pparα* (Fig 2) and consequently the elevated fatty acid β-oxidation, which led to the decreased triglyceride content (Fig 2). It is reported that AICAR-induced AMPK activation increased the content and activities of ATGL [23]javascript:newshowcontent('active','references');. We found that OM2-induced AMPK activation increased the content of ATGL as well. Our findings were in line with those reported by Gaidhu et al [23], who showed that AICAR induces AMPK activation and thus promotes energy dissipation through lipolysis, which is accompanied by up-reguation of PGC1α, PPARα and fatty acid β-oxidation. Our data also agreed with the results of several publications [41–43] in that the AMPK and PGC1α signaling axis senses the metabolic demands of cells and regulates mitochondrial function.

Fig 5 showed that the incubation of the adipocyte with siRNA of PGC1α abolished increase in ATGL expression induced by OM2, indicating that ATGL wa**s** downstream of PGC1α. Additionally, the stimulated mitochondrial biogenesis could elevate the cellular function to burn fat, which might in turn increase triglyceride lipolysis through ATGL and fatty acid oxidation. In contrast, Haemmerle et al. reported that a product of ATGL-mediated hydrolysis is required for maintaining the expression levels of PGC1α and PGC1β in the heart. But Intriguingly, the authors also observe that ATGL-mediated hydrolysis does not regulate PGC1α expression in the liver [44]. Meanwhile, it was reported that unsaturated fatty acids, EPA/DHA, up-regulated mitochondrial biogenesis and induced β-oxidation in white fat tissues [45]. Therefore, the relationship between ATGL and PGC1α is complicated. Our observations could be explained in two ways. One was that ATGL acted downstream of PGC1α in the adipocytes; another was that the existence of a feedback control of cell metabolism by ATGL and PGC1α, in that the inhibition of PGC1α would reduce mitochondria biogenesis, which would lead to mitochondrial dysfunction, and reduced mitochondrial function would result in reduced fatty acid oxidation, and subsequently accumulated free fatty acids would in turn inhibit ATGL expression in order to avoid more free fatty acid production by ATGL.

Chau et al. reported that fibroblast growth factor 21 regulated mitochondrial activities through AMPK–SIRT1-PGC1α-dependent mechanism in adipocytes [46]. Another report demonstrated that α-Lipoic acid promotes lipid metabolism by AMPK activation and SIRT1 induction in C2C12 cells [47]. Although AMPK and SIRT1 can activate in different orders [41], but we found that OM2 did not activate SIRT1 in the adipocyte at all (data not shown), which suggest that OM2 was different from fibroblast growth factor 21 and α-Lipoic acid in activating the AMPK–SIRT1-PGC1α axis in the adipocytes. In addition, Li et al. reported a similar function and mechanisms of a small molecule ZLN005 in skeletal muscles even though it has totally different structure to the oligosaccharides OM or OM2. These findings confirmed the idea of stimulating energy metabolism through PGC1α to treat metabolic syndrome, such as diabetes and obesity.

In conclusion, we showed for the first time that both oligosaccharide, OM or oligosaccharide-chromium complex, OM2, stimulated mitochondrial biogenesis, improved lipid metabolism in the adipocytes. The data obtained from our current studies indicate that OM2 had potential to be developed into a novel class of anti-diabetic medications.
